# W-doped Lanthanum Molybdenum Oxide/Lithium-Sodium-Potassium Carbonate Composite Membranes for Carbon Dioxide Permeation

**DOI:** 10.3390/ma16145128

**Published:** 2023-07-20

**Authors:** Midilane S. Medina, Sabrina G. M. Carvalho, Francisco N. Tabuti, Eliana N. S. Muccillo, Fábio C. Fonseca, Reginaldo Muccillo

**Affiliations:** 1 Center of Science and Technology of Materials, Energy and Nuclear Research Institute, Sao Paulo 05508-000, Brazil; midilanelmedina@gmail.com (M.S.M.); sabrina.carvalho@alumni.usp.br (S.G.M.C.); enavarro@usp.br (E.N.S.M.); 2 Center of Fuel Cells and Hydrogen, Energy and Nuclear Research Institute, Sao Paulo 05508-000, Brazil; fntabuti@ipen.br (F.N.T.); fabiocf@usp.br (F.C.F.)

**Keywords:** ceramic membranes, LAMOX, CO_2_ permeation, impedance spectroscopy, scanning electron microscopy

## Abstract

Single-phase tungsten-doped lanthanum molybdenum oxide (La_2_MoWO_9_) ceramic powders were synthesized using the complex polymerization technique. Porous ceramic pellets were obtained by thermally removing graphite, which served as a pore former. The porous pellets were then impregnated with molten eutectic lithium-sodium-potassium carbonates. The energy dispersive X-ray analysis and scanning electron microscopy (FEG-SEM) images of the external and fracture surfaces of the La_2_MoWO_9_-(Li,Na,K)_2_CO_3_ composite dual-phase membrane revealed the percolation of the carbonate mixture through the pores. Electrochemical impedance spectroscopy measurements conducted at temperatures below and above the melting point of the eutectic carbonate composition demonstrated the contributions of oxygen and carbonate ions to the ionic conductivity of the dual membrane. The electrical conductivity of the carbonate ions within the membrane was continuously monitored for over 1300 h with negligible degradation, implying that the membrane could be used for long-term monitoring of CO_2_ without aging effects. A comparison of FEG-SEM images taken before and after this endurance test suggested minimal fouling, indicating that the membrane could potentially replace similar zirconia- and ceria-based composite membranes.

## 1. Introduction

Lanthanum molybdate oxide (La_2_Mo_2_O_9−δ_) is a fast oxide ion conductor that exhibits a first-order reversible α-β phase transition. This transition occurs at 580 °C, causing the crystalline structure to change from monoclinic to cubic. The α-phase has a highly complex crystalline structure, with 312 crystallographically independent atoms, numerous X-ray diffraction reflections, lower symmetry, and indications of a superstructure [1]. The β-phase crystalline structure is indexed based on β-SnWO_4_, with a lattice parameter of a = 7.2014(7) Å. One key difference between these two structures is the arrangement of the oxygen atoms: β-SnWO_4_ has two fully occupied oxygen sites, while La_2_Mo_2_O_9−δ_ has three oxygen sites, with only one fully occupied and two partially occupied. The α–β phase transition leads to an increase in oxygen ion conductivity of approximately two orders of magnitude [2]. Specimens synthesized by the polymeric precursor method are phase-transition-dependent on the powder’s average particle size and ionic-conductivity-dependent on the sintering conditions [3]. However, the change in the crystalline structure adversely affects the mechanical properties and thermal stability [4]. Moreover, La_2_Mo_2_O_9−δ_ is thermodynamically unstable at low oxygen partial pressures (p_O2_) due to the reducibility of Mo^6+^, resulting in the formation of phases with partial n-type conductivity and amorphization [4,5]. To address these issues, several studies have focused on cation substitutions (W [6,7,8,9,10,11], Nd [6], Ca [12], Ba [12,13], Sr [12,14], Y [15], Cr [7,14]) for La or Mo (LAMOX family). These substitutions aim to inhibit the phase transition, stabilize the β-phase, and improve the thermomechanical stability [16]. Substituting tungsten for molybdenum has been identified as an effective strategy to stabilize the cubic phase of La_2_Mo_2_O_9−δ_ at room temperature while preserving its ionic conductivity [8]. In addition to stabilizing the β-phase at room temperature, the La_2_Mo_2−y_W_y_O_9−δ_ series also exhibits enhanced chemical stability under low p_O2_, resulting in a reduction in the formation of phases with n-type conduction as the W content increases. Although substitutions at both the La and Mo sites lead to a decrease in the ionic conductivity due to long-range vacancy ordering, the substitution of Mo for W [8] results in a structurally and chemically stable W-LAMOX compound that is suitable for various applications.

Nowadays, human activities have significant negative impacts on ecosystems due to the extensive emission of greenhouse gases. Among these gases, carbon dioxide (CO_2_) has emerged as a major global environmental concern due to its substantial release from industrial activities and the combustion of fossil fuels. To address this issue, various approaches are being pursued to develop new technologies for selective CO_2_ separation or storage.

Several types of porous membranes utilizing polymers [17], silica [18], and zeolites [19] have been tested for CO_2_ separation, but their use is limited by the low operating temperatures (up to 300 °C) required [20]. A dual-phase composite membrane that combines a stainless-steel porous support with a eutectic mixture of molten carbonates, exhibiting high permeation in the temperature range of 450–650 °C, has been proposed [21]. However, at temperatures exceeding 650 °C, low-conductivity phases are formed at the metal surface, inhibiting CO_2_ permeation. Additionally, achieving pure CO_2_ separation requires an additional step to remove oxygen (O_2_) from the CO_2_ permeate, making the process expensive. To overcome these challenges, the use of porous supports based on ionic conducting ceramics or mixed-conducting ceramics has been proposed [22]. Dual-phase composite ceramic membranes are considered a promising option for achieving efficient and cost-effective CO_2_ separation.

In a dual-phase membrane, a porous solid electrolyte ceramic support is filled with a eutectic mixture of binary or ternary alkali carbonates. Selective CO_2_ separation occurs through the transport of carbonate ions in the carbonate phase and oxygen ions in the ceramic phase. The performance of CO_2_ permeation is limited by the oxygen ion conductivity of the solid electrolyte [23]. Therefore, optimizing the operation of these membranes relies on achieving high ionic conductivity in the solid electrolyte at the melting point of the carbonate phase.

In addition to high ionic conductivity, an ideal dual-phase membrane for CO_2_ separation should possess properties such as thermal and chemical stability, as well as CO_2_ selectivity and permeability. Various compounds, including doped lanthanum strontium cobaltite [24] and ceria-based [25,26] compounds, have been proposed to meet these requirements. However, some of these compounds encounter issues with structural phase transformations due to the reaction between CO_2_ and the ceramic phase [21]. Therefore, enhancing the oxygen conductivity of ceramics and discovering new compounds have been the focus of numerous research studies.

In this context, W-LAMOX has emerged as a promising candidate for use as a porous ceramic support due to its desirable characteristics. Despite the decrease in ionic conductivity resulting from the substitution of W for Mo [9], the achieved structural and chemical stability make W-LAMOX suitable for carbonate dual-phase membranes used in CO_2_ separation, considering that this application necessitates chemically and thermally stable ceramics with high oxygen ion conductivity.

This work describes the synthesis, structural characterization, and electrical properties of W-doped lanthanum molybdenum oxide (W-LAMOX), as well as dual-phase W-LAMOX/(Li-Na-K)_2_CO_3_ membranes. Furthermore, the long-term stability of carbon dioxide ion conduction at the melting point of the eutectic lithium-sodium-potassium carbonate is investigated.

## 2. Materials and Methods

### 2.1. W-LAMOX Synthesis; Membrane Preparation

W-doped lanthanum molybdenum oxide (La_2_MoWO_9_, hereafter W-LAMOX) was synthesized using the complex polymerization method. The following chemicals were used: molybdenum (VI) oxide (MoO_3_ 99%, Sigma Aldrich, St. Louis, MO, USA), tungsten (VI) oxide (WO_3_ 99.8%, Sigma Aldrich), lanthanum (III) nitrate hexahydrate (La(NO_3_)_3_·6H_2_O 99.999%, Sigma Aldrich), citric acid (C_6_H_8_O_7_ 99.5%, Synth, Colorado Springs, CO, USA), ethylene glycol (C_2_H_4_(OH)_2_, Vetec, Speyer am Rhein, Germany), hydrogen peroxide (H_2_O_2_ 30%, Nalgon, Golden Lake, ON, Canada), and ammonium hydroxide (NH_4_OH 28%, CRQ, Carlsbad, CA, USA).

The synthesis process involved several steps: (1) dissolution of stoichiometric amounts of MoO_3_ and WO_3_ in separate solutions of H_2_O_2_ and NH_4_O_4_ (5 vol.%) at room temperature (for MoO_3_) and at 200 °C (for WO_3_); (2) dissolution of La(NO_3_)_3_·6H_2_O in distilled water at room temperature; and (3) mixing the metal precursor solutions with a solution of citric acid at room temperature. The citric acid/metals molar ratio was fixed at 2:1; (4) stirring the mixture and increasing the temperature to 100 °C; (5) the addition of ethylene glycol to promote citrate polymerization, with a 60:40 citric acid/ethylene glycol mass ratio; (6) continuous stirring at 100 °C until the solution turned into a gel; and (7) heating the gel at a rate of 5 °C/min for precalcination at 200 °C for 2 h and calcination at 550 °C for 4 h.

Dense W-LAMOX pellets with a cylindrical shape (14 mm diameter and 3 mm thickness) were obtained by uniaxial cold pressing at 70 MPa (Skay, São Paulo, Brazil), followed by isostatic pressing at 200 MPa (National Forge Co., Irvine, PA, USA) in vacuum-sealed plastic bags. The pellets were then sintered at 1050 °C for 2 h. Porous W-LAMOX was obtained by incorporating ~10 wt.% graphite (Micrograf, 99507 grade, Nacional de Grafite, Itapecerica, Brazil) as a sacrificial pore former. The mixture was cold pressed, and the green pellets were sintered at ~800 °C for 2 h to remove the graphite, followed by sintering at 1000 °C with heating and cooling rates of 5 °C/min. The resulting porous W-LAMOX was impregnated with a eutectic mixture of lithium carbonate (Li_2_CO_3_), sodium carbonate (Na_2_CO_3_), and potassium carbonate (K_2_CO_3_) in a molar ratio of 43.5:31.5:25.0 (hereafter, LNKC). The impregnation process involved placing an LNKC pellet on top of the porous W-LAMOX, heating it to 500 °C at a rate of 5 °C/min, and maintaining that temperature for 1 h. After cooling, any residual carbonates were removed from the W-LAMOX surfaces. The skeletal density of the sintered porous W-LAMOX was determined using the Archimedes method in a Mettler Toledo AG245 analytical balance (Columbus, OH, USA).

### 2.2. Thermal, Structural, and Electrical Analyses

DTATG experiments were performed in a W-LAMOX/graphite mixture with STA 409E equipment (Netzsch, Selb, Germany) from room temperature to 1000 °C at a rate of 5 °C/min under flowing synthetic air at 10 L min^−1^.

X-ray diffraction data from the W-LAMOX samples (powder and pellets) were collected in a D8 Advance diffractometer (Bruker-AXS, Karlsruhe, Germany) with a θ-θ Bragg-Brentano configuration and 40 kV-40 mA Cu-k_α_ radiation in the 20°–80° 2θ range with a 0.05° step size and 3 s per step.

The images of the surfaces of the porous W-LAMOX were observed before and after impregnation with LNKC with a scanning electron microscope (FEG-SEM Inspect F50, FEI, Brno, Czech Republic). Energy dispersive X-ray spectroscopy (EDX) analyses were performed at both parallel and fracture surfaces of the impregnated samples to evaluate the permeation of the carbonate phase through the pores and its presence in the bulk of the samples.

The electrical conductivity of both porous and LNKC-impregnated W-LAMOX was evaluated with an impedance analyzer (HP 4192A, Yokogawa-Hewlett Packard, Tokyo, Japan) connected to a desktop workstation (series 360 HP Controller) from 300 °C to 650 °C, using thin gold disks as electrodes; [−Z″(ω) × Z′(ω)] data (Z″ and Z′ are the imaginary and the real components of the electrical impedance, respectively; ω = 2πf, f is the frequency of the applied bias) were collected in the 5 Hz–13 MHz frequency range with 20 points per decade, under a 200 mV input signal, with special software [27]. For the measurements, the sample was spring-loaded in a homemade alumina sample chamber with 1 m platinum leads connected to the impedance analyzer using the HP16047C test fixture; the temperature was monitored with a type K (chromel-alumel) thermocouple with its sensing tip positioned close to the sample. For long-term electrical conductivity tests, approximately 100 impedance spectroscopy diagrams were collected over >1300 h in an impregnated sample in the sample chamber inside a Lindberg-Blue M (Lindberg, Watertown, NY, USA) furnace kept at 480 °C.

## 3. Results and Discussion

### 3.1. Dense W-LAMOX

**Figure 1:**
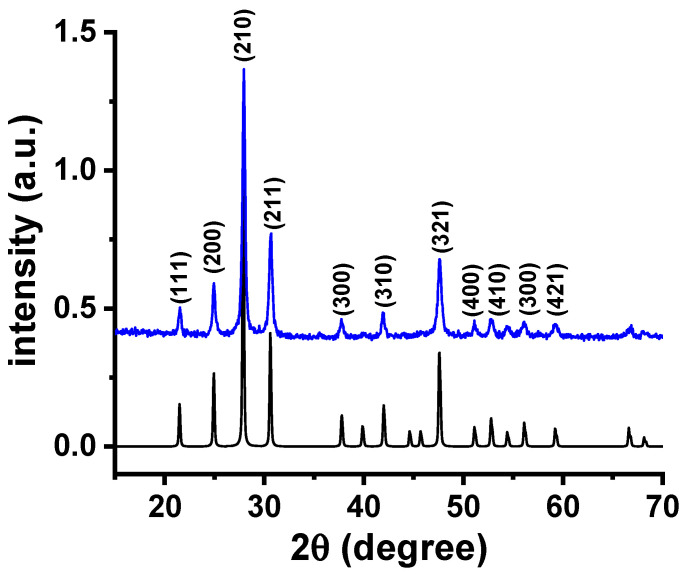
X-ray diffraction patterns for W-LAMOX; top: powder synthesized by the polymeric precursor method and calcined at 550 °C/4 h; bottom: LAMOX ICSD 172611 file.

The room temperature X-ray diffraction pattern of the W-LAMOX powder is depicted in Figure 1. La_2_Mo_2_O_9−δ_ undergoes a phase transition from the monoclinic α-phase to the cubic β-phase at 580 °C, which is characterized by a split in the (321) reflection at 2θ = 47.632° [28]. The absence of this split indicates the stabilization of the high-temperature conducting cubic phase. The Bragg reflections observed in the diffractogram shown in Figure 1 are consistent with the ICSD 172611 file, which has a lattice parameter of 7.142 Å, as determined using GSAS/EXPGUI software [29]. This agrees with the data reported for the cubic phase [8,16].

**Figure 2:**
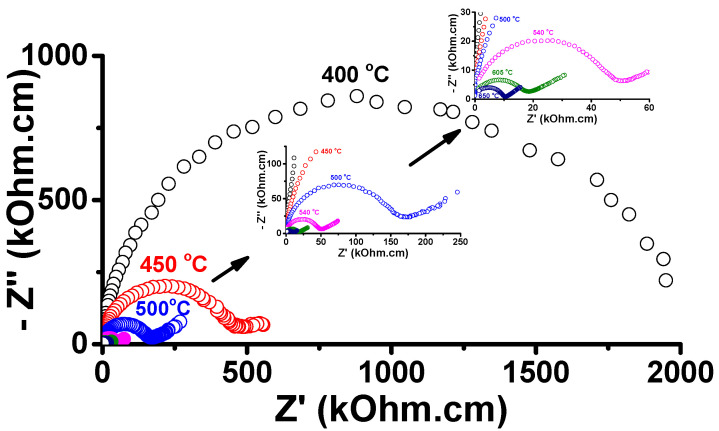
Impedance spectroscopy diagrams in the 400–650 °C range for W-LAMOX sintered at 1050 °C/2 h; insets show diagrams for higher temperatures.

**Figure 3:**
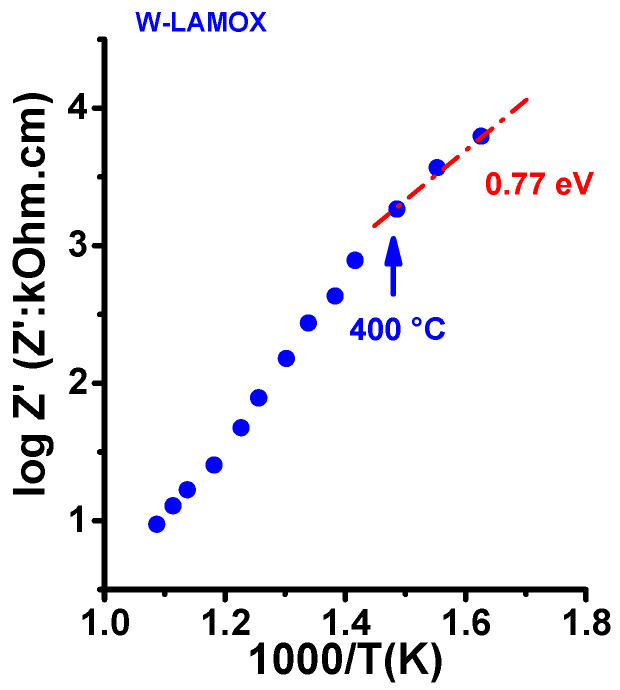
Electrical resistivity of W-LAMOX sintered at 1050 °C/2 h as a function of the reciprocal temperature.

Figure 2 shows impedance spectroscopy diagrams at various temperatures within the range of 400–650 °C. The diagrams consist of one semicircle in the high-frequency region and a spike at lower frequencies for temperatures higher than 400 °C. The semicircle is due to the contributions of the resistivity of the bulk (grains) and interfaces (mainly grain boundaries). The spike represents the resistivity due to the interaction of the charge carriers (oxygen ions) with the gold electrodes [30]. Additionally, Figure 3 displays a plot of the total electrical resistivity as a function of the reciprocal of the absolute temperature. The electrical resistivity values were determined at the intersection of the semicircles with the real axis in the low-frequency region of the [−Z″(ω) × Z′(ω)] impedance diagram. At approximately 400 °C, the compositions of the LAMOX family show modification of the ionic conductivity behavior, from the Arrhenius to the Vogel–Tamman–Fulcher (VTF) type [8]. The activation energy of 0.77 eV reported for the ionic conductivity is close to reported values for W-LAMOX [8]. 

### 3.2. Porous W-LAMOX

The skeletal density of the sintered porous W-LAMOX, determined using the Archimedes method in a Mettler Toledo AG245 analytical balance (Columbus, OH, USA), was approximately 60% of the theoretical density [8].

**Figure 4:**
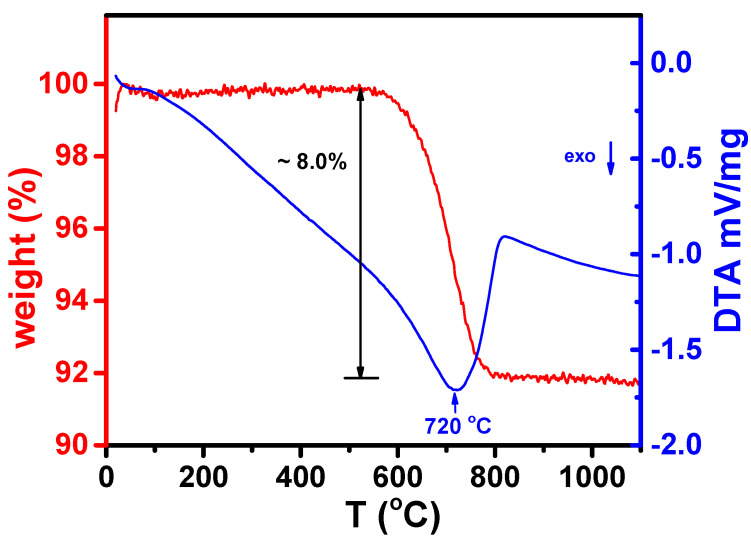
Thermogravimetric and differential thermal analysis curves of W-LAMOX mixed with 10 wt.% graphite (sacrificial pore former).

Figure 4 illustrates the results of the DTATG analysis performed from room temperature to 1100 °C on a mixture of W-LAMOX with 10% graphite.

The DTATG curves exhibit the thermal decomposition behavior of the mixture, revealing an exothermic peak at around 720 °C, accompanied by an approximately 8% weight loss, attributed mostly to the thermal removal of graphite. Beyond 800 °C, the weight loss becomes negligible, indicating the absence of residual graphite in the composite. Consequently, a temperature of 800 °C was applied to eliminate graphite and obtain porous samples.

**Figure 5:**
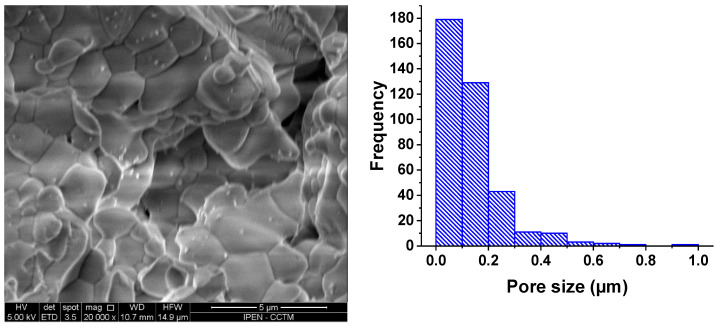
(**a**) Scanning electron microscopy micrographs of the porous W-LAMOX pellet surface; (**b**) distribution of pore sizes determined using ImageJ software [31].

Figure 5a displays a scanning electron microscopy (SEM) micrograph depicting a typical surface of a W-LAMOX pellet that was sintered with 10 wt.% graphite. Figure 5b illustrates the pore size distribution, which was analyzed using ImageJ software [31]. The sample exhibits consistent and uniformly distributed pore sizes, with an average pore size of approximately 0.21 µm. The opposite parallel surface and fracture surfaces of these ceramic pellets exhibit similar pore structures.

### 3.3. W-LAMOX/(Li,Na,K)_2_CO_3_ Composite Membrane

**Figure 6:**
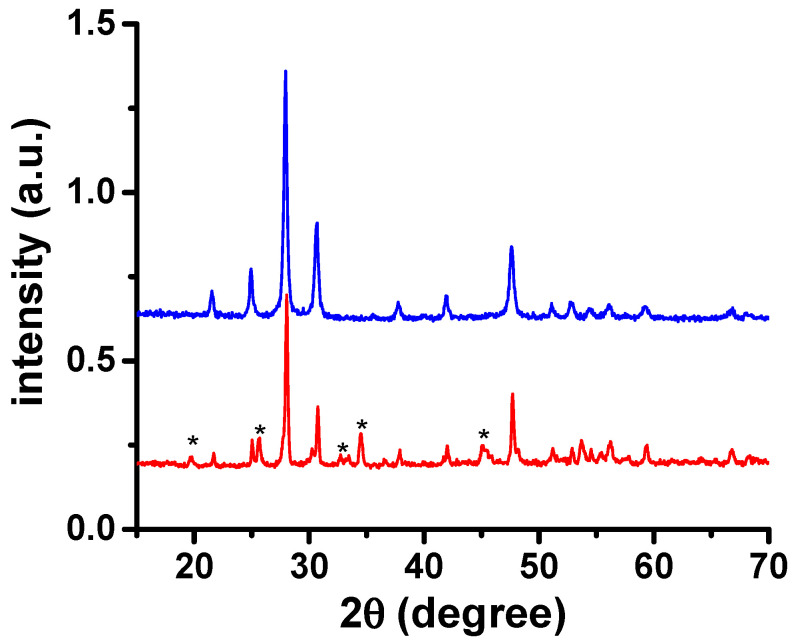
X-ray diffraction patterns of porous W-LAMOX (blue curve) and porous W-LAMOX impregnated with LNKC (red curve). The asterisks indicate LNKC reflections.

Figure 6 shows the diffraction pattern of the W-LAMOX/(Li,Na,K)_2_CO_3_ composite membrane. Upon comparison with the X-ray diffraction pattern of W-LAMOX, no significant changes were observed in the Bragg reflections. This suggests that there were no apparent reactions between W-LAMOX and molten LNKC. However, the presence of certain peaks marked with asterisks in the diffractogram, attributed to LNKC, indicate its presence in the composite membrane.

**Figure 7:**
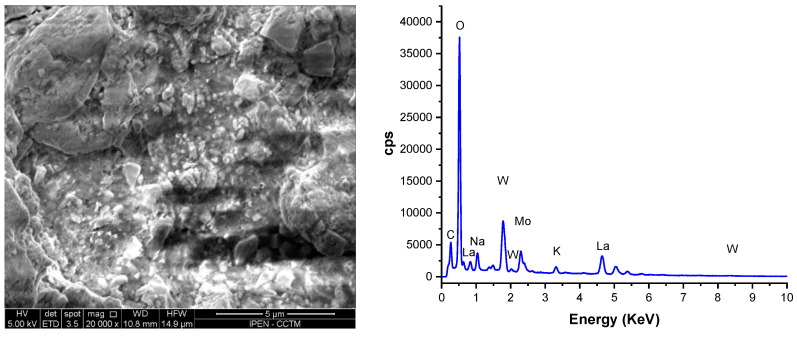

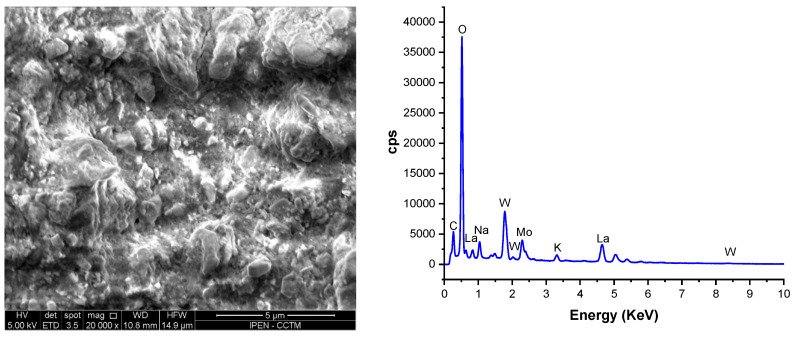
Scanning electron microscopy micrographs (**left**) and EDX plots (**right**) of the two parallel surfaces of W-LAMOX/LNKC.

Figure 7 exhibits scanning electron microscopy (SEM) micrographs and energy-dispersive X-ray spectroscopy (EDX) plots obtained from data collected from both surfaces of the composite membrane. Specifically, these images and plots capture the porous structure of the W-LAMOX pellet following thermal infiltration with the (Li,Na,K)_2_CO_3_ eutectic composition.

The images reveal that the composite membrane features a dense, solid electrolyte matrix, partially covered by carbonates. The matrix effectively fills the entire porous network. EDX analyses provided evidence that sodium (Na) and potassium (K) are present on both surfaces, indicating that molten LNKC successfully permeated the interconnected porous structure during the impregnation procedure. This suggests thorough infiltration of the composite membrane by the molten LNKC electrolyte.

**Figure 8:**
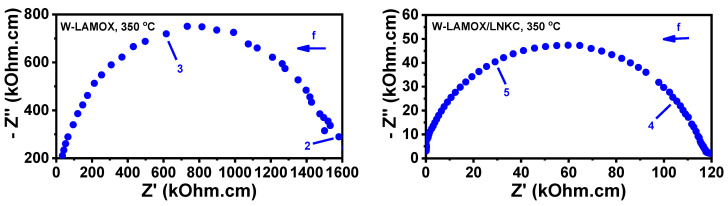

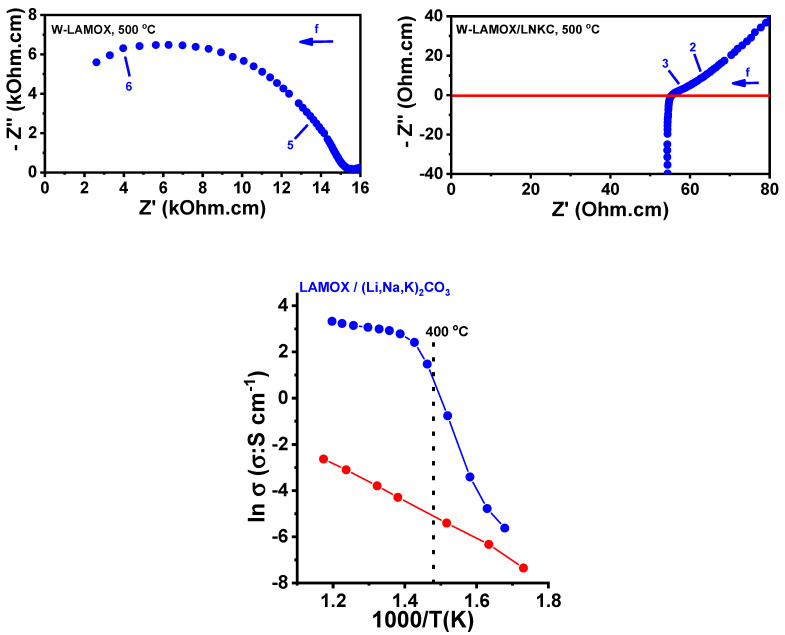
Top: Impedance spectroscopy diagrams of porous W-LAMOX (**left**) and W-LAMOX/LNKC (**right**) at 350 °C (**top**) and 500 °C (**bottom**); numbers stand for log f (f: Hz). bottom: Arrhenius plots of the W-LAMOX porous matrix (red curve) and W-LAMOX/LNKC (blue curve).

Figure 8 shows the impedance spectroscopy diagrams of the porous W-LAMOX (left) and W-LAMOX/LNKC (right) samples, measured at 350 °C (top) and 500 °C (bottom). At 350 °C, both samples exhibit apparently single-arc impedance diagrams without clear differentiation between the electrical resistivity contributions of grain and interfaces (mainly grain boundaries). Moreover, the total electrical resistivity of W-LAMOX/LNKC is approximately one order of magnitude lower than that of porous W-LAMOX, indicating that filling the pores with LNKC leads to an enhancement of the total conductivity of the W-LAMOX/LNKC composite. At 500 °C, the impedance diagram of the porous matrix remains a single arc, while the diagram of W-LAMOX/LNKC changes its shape due to the contribution of the inductance of the coaxial cables [32] and exhibits enhanced conductivity above the melting temperature of LNKC (396 °C) [33]. This indicates that the conductivity at this temperature is improved by the contribution of the carbon dioxide ion conductivity of the molten LNKC second phase within the porous structure of the composite membrane.

In the LNKC-impregnated porous matrix, the Arrhenius plot displays three distinct regions: (1) for temperatures below approximately 370 °C, the electrical conductivity exhibited linear behavior with an activation energy of 0.77 eV, which is close to previously reported values for W-LAMOX. The conductivity in this temperature range is attributed to thermally activated oxygen ion conductivity [1,2]. (2) In the subsequent region, the electrical conductivity demonstrated a significant increase, primarily driven by the contribution of carbon dioxide ions, resulting from the onset of LNKC melting [34]. (3) At temperatures exceeding 400 °C, the linear behavior resumed due to the dominant contribution of electrical conductivity from carbon dioxide ions in the molten state of the LNKC. This region exhibited a typical activation energy of 0.19 eV, representative of the electrical conductivity behavior observed [35,36,37]. 

**Figure 9:**
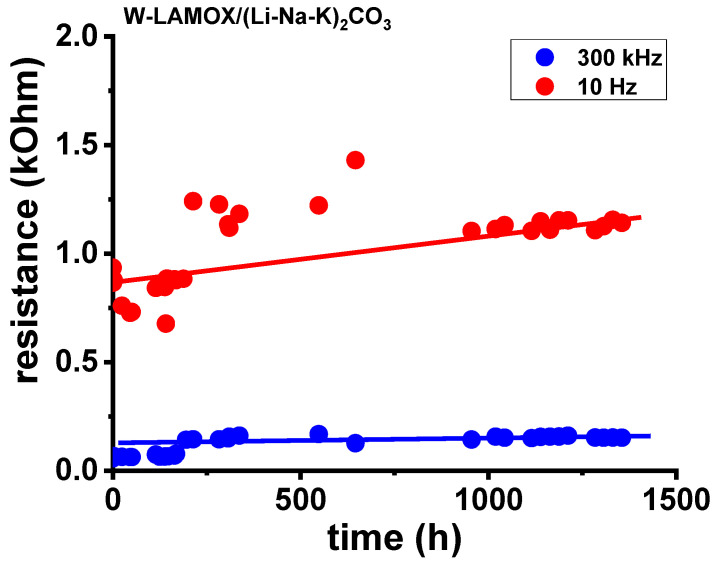
Electrical resistance long-term operational stability data collected at 300 kHz and 100 Hz at 480 °C in W-LAMOX/LNKC.

Figure 9 presents the electrical resistance values of W-LAMOX/LNKC ceramic membranes plotted as a function of the time that the membrane was kept inside the sample chamber of the furnace at 480 °C. The measurements were taken at two frequencies: one at the region containing the bulk contribution (300 kHz) and the other containing the electrode contribution (10 Hz). The bulk resistance remained constant, indicating that the membrane did not undergo any degradation. However, the interface resistance showed an increase over time, likely attributed to the oxidation of the electrodes. It is important to note that these measurements were conducted in an air environment.

**Figure 10:**
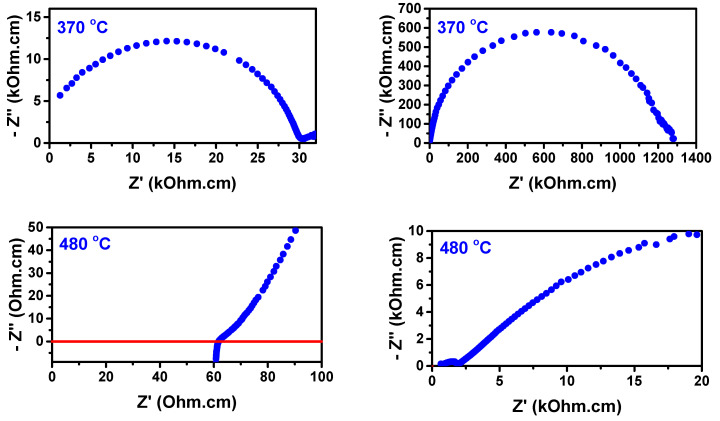

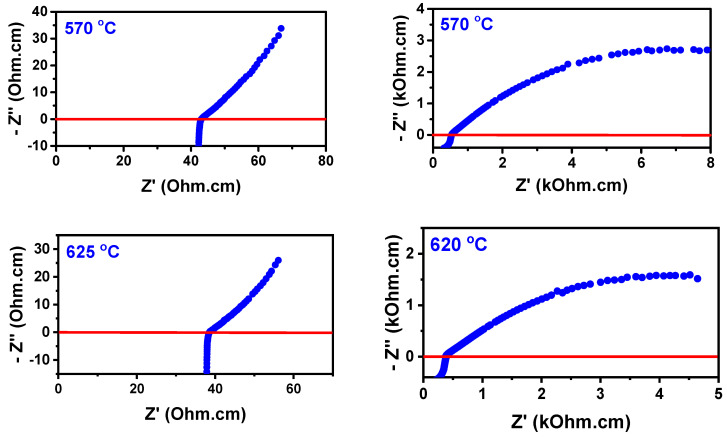
Impedance spectroscopy diagrams of W-LAMOX/LNKC before (**left**) and after (**right**) ~1300 h at 480 °C.

Impedance spectroscopy measurements were conducted on the W-LAMOX/LNKC dual-phase composite membranes, both before and after a long-term annealing period of approximately 1300 h at 480 °C. The measurements were performed at various temperatures, including those below and above the melting point of the carbonate phase. Figure 10 displays a selection of impedance spectroscopy diagrams obtained from these measurements.

**Figure 11:**
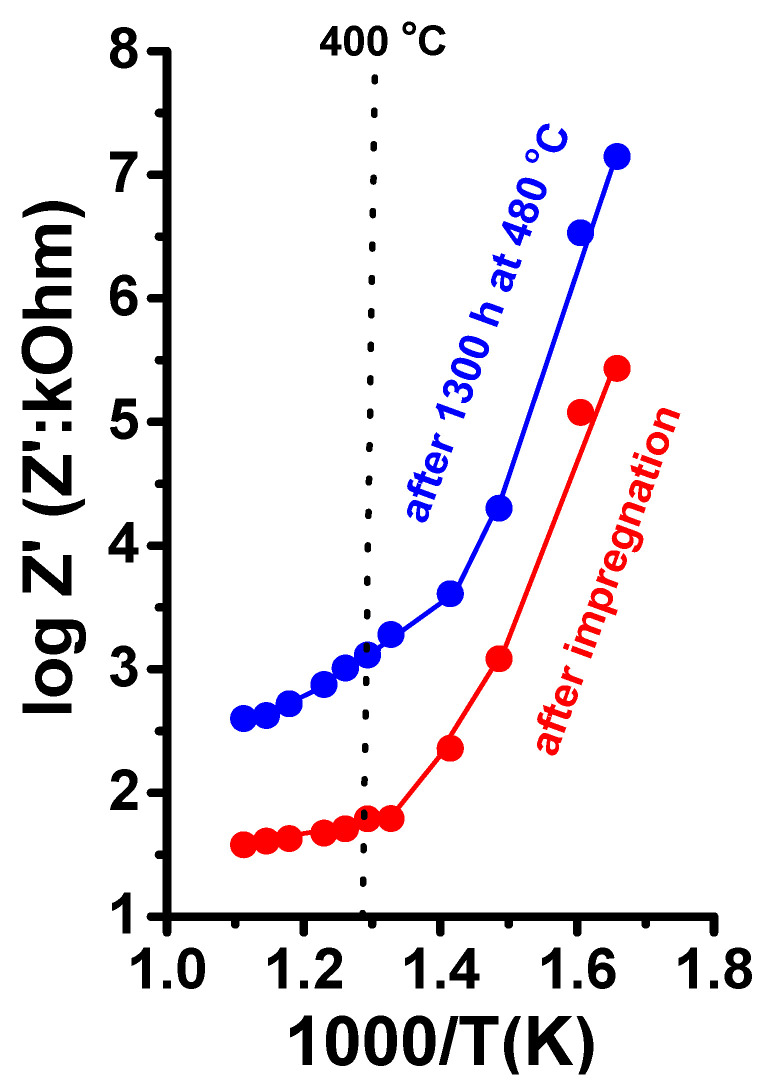
Arrhenius plots of the W-LAMOX/LNKC ceramic membrane before and after being kept at 480 °C for ~1300 h.

A noteworthy observation in the impedance spectroscopy measurements is the increase in the electrical resistivity of approximately one order of magnitude. This increase is attributed to the partial oxidation of the electrodes at the interface between the electrodes and the specimen. The impact of this oxidation is evident in the Arrhenius plots of the electrical resistivity, as depicted in Figure 11. The temperature of 400 °C, near the melting point of LNKC, is signaled.

**Figure 12:**
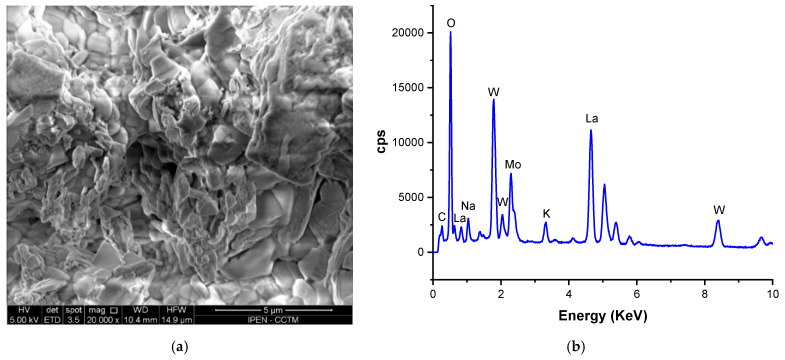
(**a**) SEM micrographs and (**b**) EDX analysis of the W-LAMOX/LNKC ceramic membrane before and after being kept at 480 °C for ~1300 h.

The surface of the W-LAMOX/LNKC ceramic membrane was examined using a scanning electron microscope before and after the long-term electrical measurements at 480 °C. Figure 12a displays the corresponding image, while Figure 12b presents the results of the EDX analysis.

Based on the image and the EDX elemental analysis, it is evident that the carbonates remained present in the sample of the W-LAMOX/LNKC ceramic membrane. The EDX analysis confirmed the detection of sodium and potassium elements, indicating their presence in the composite. However, it is important to note that lithium was not detected by the EDX technique in this analysis.

## 4. Conclusions

Lanthanum molybdenum oxide doped with tungsten (La_2_MoWO_9_, W-LAMOX) ceramic powder was successfully synthesized by the complex polymerization method. Porous W-LAMOX ceramic pellets were prepared by the thermal removal of graphite powder, which was added as sacrificial pore former. Molten eutectic compositions of lithium, sodium, and potassium carbonates were impregnated in the porous pellets. The electrical resistivity and the surfaces of both porous and impregnated samples were analyzed by the impedance spectroscopy and scanning electron microscopy (SEM) techniques, respectively. The SEM analyses showed that the carbonate phase percolated through the bulk of the pellets. Analysis of the carbon dioxide ion resistivity in the molten state for more than 1300 h showed negligible degradation, providing evidence of the chemical resistance of W-LAMOX to the molten carbonates. W-LAMOX is thus proposed as a solid electrolyte matrix for ceramic membranes for application either in carbon dioxide capture or as a carbon dioxide sensor.

## Data Availability

Not applicable.
